# Assessing the Effectiveness of Antibiotic Therapy Against Common Gram-Negative Bacteria in a Saudi Arabian Hospital Using the Drug Resistance Index

**DOI:** 10.7759/cureus.22168

**Published:** 2022-02-13

**Authors:** Muhammad Yaseen, Abdulhakeem Althaqafi, Fayssal Farahat, Asim Alsaedi, Abdulfattah Mowallad, Eili Klein, Katie Tseng, Sabiha Essack

**Affiliations:** 1 Infection Prevention and Control, Velindre University National Health Service Trust, Cardiff, GBR; 2 Infectious Diseases, King Abdulaziz Medical City, King Saud Bin Abdulaziz University for Health Sciences, King Abdullah International Medical Research Center, Jeddah, SAU; 3 Infection Prevention and Control, King Abdulaziz Medical City, King Saud Bin Abdulaziz University for Health Sciences, King Abdullah International Medical Research Center, Riyadh, SAU; 4 Infectious Diseases and Control, King Abdulaziz Medical City, Jeddah, SAU; 5 Pathology and Laboratory Medicine, King Abdulaziz Medical City, Jeddah, SAU; 6 Emergency Medicine, Center For Disease Dynamics, Economics & Policy, Washington, DC, USA; 7 Research and Development, Center For Disease Dynamics, Economics & Policy, Washington, DC, USA; 8 Pharmacology and Therapeutics, College of Health Sciences, University of KwaZulu-Natal, Durban, ZAF

**Keywords:** antibiotic consumption, saudi arabia, interventions, intensive care unit, antibiotic resistance, drug resistance index

## Abstract

Introduction: Assessing the effectiveness of antibiotics and communicating the problem of resistance are essential when devising antimicrobial stewardship programs (ASPs) in hospital settings. The Drug Resistance Index (DRI) is a useful tool that combines antibiotic consumption and bacterial resistance into a single measure. In this study, we used the DRI to assess the impact of introducing a new antibiotic restriction form on antibiotic effectiveness for the treatment of Gram-negative infections in the intensive care unit (ICU).

Methods: This was an observational study to assess and evaluate the antibiotic susceptibility of Gram-negative bacteria and antibiotic prescribing rates for the antibiotics indicated for Gram-negative bacteria following the introduction of a new antibiotic restriction form. The study was conducted from 2015 to 2017 at King Abdulaziz Medical City, a tertiary care facility in Jeddah, Saudi Arabia. Changes in antibiotic effectiveness before and after the introduction of the form were evaluated by calculating the DRI for four of the most common Gram-negative pathogens and eight commonly used antibiotic classes.

Results: The overall DRI for the adult ICU was higher (59.45) in comparison to the hospital-wide DRI (47.96). A higher DRI was evident for carbapenems and antipseudomonal penicillins + beta-lactamase inhibitors. *Acinetobacter baumannii* had the highest DRI followed by *Klebsiella pneumoniae* in both the adult ICU and hospital-wide. After the implementation of antibiotic restriction in the adult ICU, the DRI for carbapenems was significantly lower in the post-intervention phase (from 31.61 to 26.05) (p = 0.031).

Conclusion: The DRI is a useful tool for tracking the effectiveness of antibiotics over time. The results of our study are significant in the way that it highlights the importance of having an effective antibiotic stewardship program in healthcare settings and regular feedback of antibiotic consumption data to the stakeholders to keep the antibiotic prescriptions in check, thereby ensuring their sustained effectiveness.

## Introduction

Antibiotics are useful agents against common infectious diseases and have proven to save many lives since their invention. However, antibiotic resistance, driven in part by the overuse and inappropriate prescribing of antibiotics [1], has become a public health issue for clinicians worldwide. The treatment options for patients infected with multidrug-resistant (MDR) bacteria are increasingly limited and nonexistent in cases where bacteria are resistant to all available antibiotics. Consequently, MDR infec­tions are associated with higher morbidity and mortality, as well as longer hospital stays [2,3], particularly in intensive care units (ICUs) where antibiotic use is widespread and resistance among Gram-negative bacteria is high [4].

In Saudi Arabia, several research studies have found a predominance of MDR infections caused by Gram-negative bacteria, including *Acinetobacter baumannii*,* Pseudomonas aeruginosa*,* Klebsiella pneumoniae*, and *Escherichia coli* [5-8]. Characterized by their growing resistance to carbapenems and third-generation cephalosporins, these organisms are among the list of priority pathogens of the World Health Organization (WHO) for which research and development of new antibiotics are urgently needed [9]. Monitoring the trends in resistance for these bacteria is essential for assessing the overall burden of resistance over time and developing local empiric treatment guidelines for bacterial infections. However, communicating the problem of resistance to policymakers and clinicians in a way that is easy to understand can be challenging, given that infections can be caused by a broad spectrum of pathogens and treated with numerous drugs.

To address these challenges, the Drug Resistance Index (DRI) was created as a simple and effective means of communicating gaps in antibiotic effectiveness. Similar to stock market indices computed from market valuations across companies of comparable sizes, the DRI aggregates measurements of antibiotic resistance and consumption across multiple drug-pathogen combinations into a single measure. The simple metric can then be used to compare changes in antibiotic effectiveness over time and across geographic settings [10]. In this study, we assessed the effectiveness of a set of antibiotics used to treat common Gram-negative bacterial infections in both the ICU and hospital-wide by calculating their DRI at various time points. We also use the DRI to evaluate the impact of introducing an antibiotic restriction form on antibiotic effectiveness in the adult ICU. To the best of our knowledge, this is the first study in Saudi Arabia to calculate the DRI for a healthcare facility in the Middle East region.

Monitoring antibiotic consumption and resistance and sharing results with stakeholders, especially prescribers, can guide policy toward more effective antibiotic usage. However, it is a challenge to present this information to policymakers and prescribing physicians in a way that is easily understandable when the data on antibiotic resistance and antibiotic consumption is presented separately. The DRI can bridge this gap by presenting this information in a single measure that is easily understandable. The index can also be useful when trying to compare between different geographic settings and types of infections and indicating changes in antibiotic effectiveness over time [10].

## Materials and methods

This was an observational study to assess and evaluate the antibiotic susceptibility of Gram-negative bacteria and antibiotic prescribing rates for the antibiotics indicated for Gram-negative bacteria following the introduction of a new antibiotic restriction form. The study was conducted from January 2015 to December 2017 at King Abdulaziz Medical City, a tertiary care hospital located in the city of Jeddah in the Western region of Saudi Arabia. King Abdulaziz Medical City, Jeddah, is an over 500-bed tertiary care hospital serving the National Guards and their eligible dependents in the Western Region of Saudi Arabia. The adult ICU is a combined unit of medical and surgical patients with a bed capacity of 28 beds.

In July of 2016, an antibiotic restriction form was introduced to the adult ICU as part of the hospital’s antimicrobial stewardship program (ASP). The form required physicians to get approval for an antibiotic therapy from either the clinical pharmacist or an infectious disease physician assigned to the antimicrobial stewardship program (ASP) prior to prescribing. To evaluate the impact of implementing antibiotic restriction on drug effectiveness in the ICU using the DRI, data on antibiotic prescribing and resistance was obtained from the adult ICU, in addition to facility-wide data.

The DRI was calculated using the formula introduced by Laxminarayan and Klugman (2011): 𝐷𝑅𝐼 = ∑kptk𝑞tk, where ptk is the proportion of resistance among all included pathogens to drug k for time t and 𝑞tk is the proportion of drug k used for the treatment of those pathogens in all drugs included in the index for time t. The resulting score is between 0 and 100, where 0 indicates 100% susceptibility and 100 indicates 100% resistance [10]. The time unit used for this study was a quarter or overall period of three years (2015-2017) in some cases.

The organisms included in the study were *A. baumannii*, *K. pneumoniae*, *P. aeruginosa*, and *E. coli*. These organisms were isolated from routine clinical samples sent from inpatient wards/units and obtained from patients suffering from infections of the blood­stream, respiratory tract, skin and soft tissue, or urinary tract. Eight classes of antibiotics commonly used in the treatment of the aforementioned Gram-negative infections were included in the study: aminoglycosides (e.g., amikacin and gentamicin), carbapenems (e.g., imipenem and meropenem), third- and fourth-generation cephalosporins (e.g., cefepime, ceftazidime, and ceftriaxone), fluoroquinolones (e.g., ciprofloxacin), glycylcyclines (e.g., tigecycline), penicillins + beta-lactamase inhibitors (e.g., amoxicillin-clavulanate and ampicillin-sulbactam), antipseudomonal penicillins + beta-lactamase inhibitors (e.g., piperacillin-tazobactam), and polymyxins (e.g., colistin).

Data were collected for three years from January 2015 to December 2017. Resistance data based on antimicrobial susceptibility tests were extracted from the Cerner Millennium software used for ordering and reporting all microbiology laboratory results. Antibiotic consumption data were extracted by the Information Services from the electronic QuadraMed software used by hospital physicians and pharmacists for prescribing and dispensing medications. The number of antibiotics consumed was measured based on days of therapy (DOT), defined as the sum of days for which any amount of antibiotics was administered to individual patients regardless of dose and standardized over 1,000 patient days. To calculate the DOT, patient admissions and discharge data were provided by the Bed Management. Patient days were calculated based on the number of patients admitted to the hospital for any portion of each day (including admission and discharge days) during the study period irrespective of antibacterial consumption. Data analysis was conducted using the statistical software IBM SPSS. T-test analyses were conducted to identify whether increasing or decreasing trends in the DRI were statistically significant at the 5% level.

## Results

**Figure 1:**
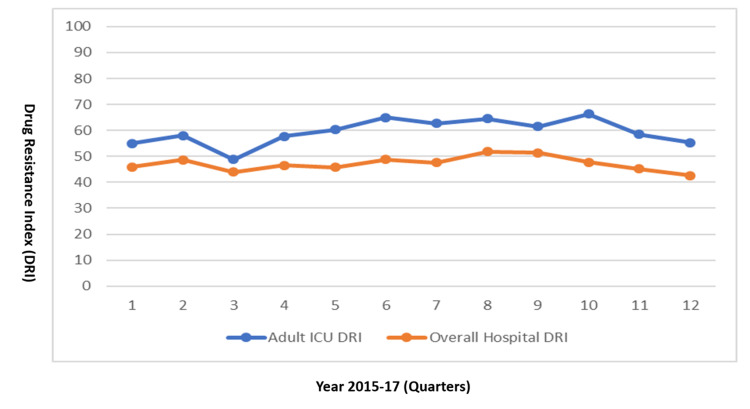
Overall Drug Resistance Index, hospital-wide versus adult ICU (2015–2017)

**Figure 2:**
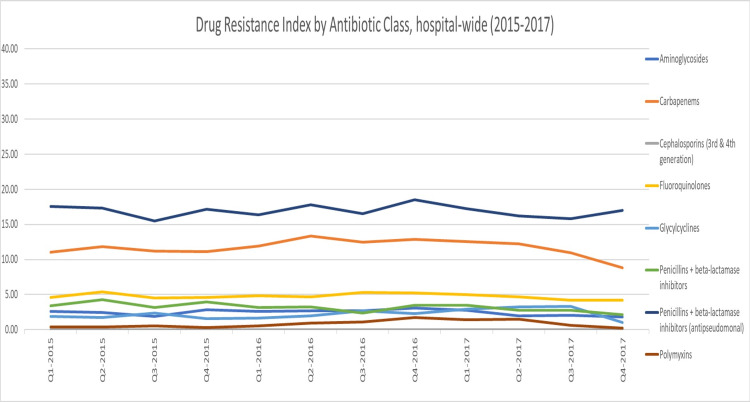
Drug Resistance Index by antibiotic class, hospital-wide (2015–2017)

**Figure 3:**
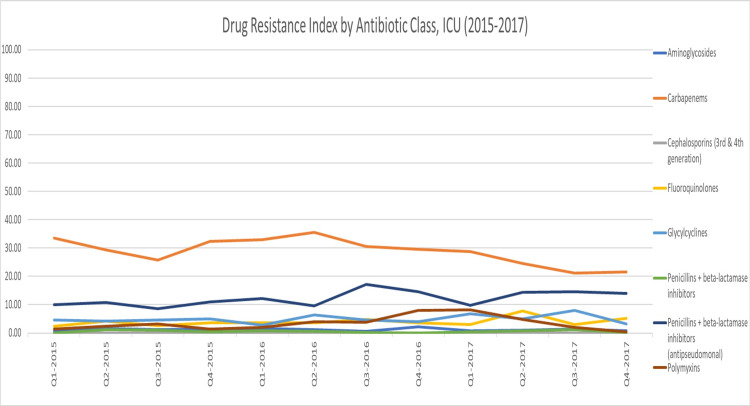
Drug Resistance Index by antibiotic class, adult ICU (2015–2017)

**Figure 4:**
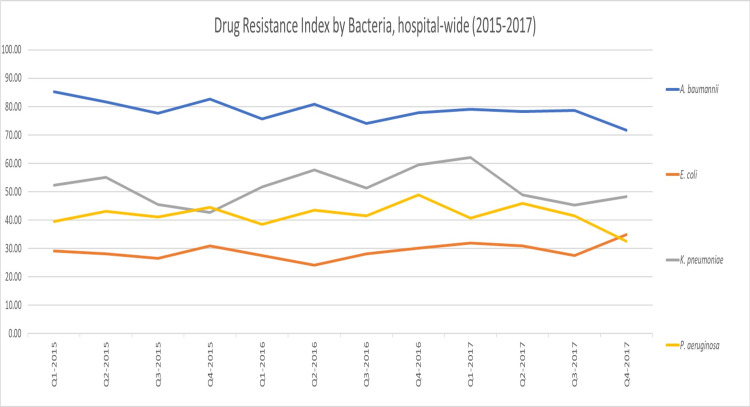
Drug Resistance Index by bacteria, hospital-wide (2015–2017)

**Figure 5:**
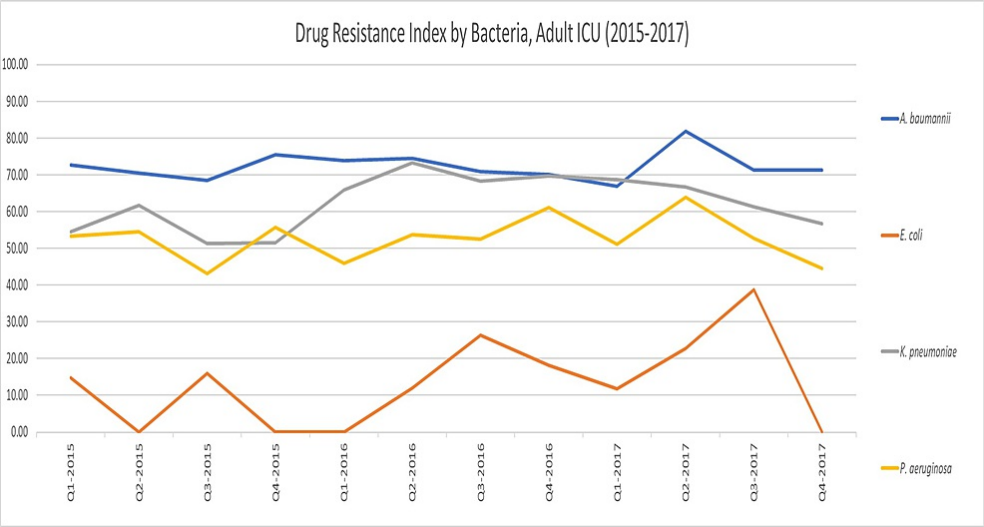
Drug Resistance Index by bacteria, adult ICU (2015–2017)

Compared to the hospital-wide DRI (47.96; cov = 6.65%), the average DRI for the adult ICU was higher (59.45), as shown in Figure 1. Examining the DRI by drug class, we found that antipseudomonal penicillins + beta-lactamase inhibitors and then carbapenems had the highest DRI hospital-wide (16.90 and 11.69, respectively), while in the adult ICU, carbapenems had the highest DRI (28.83), followed by antipseudomonal penicillins + beta-lactamase inhibitors (12.27) (Figure 2 and Figure 3). At the pathogen level, in both the adult ICU and hospital-wide, *A. baumannii* had the highest DRI, followed by *K. pneumoniae*​​​​​​ (Figure 4 and Figure 5).

**Figure 6:**
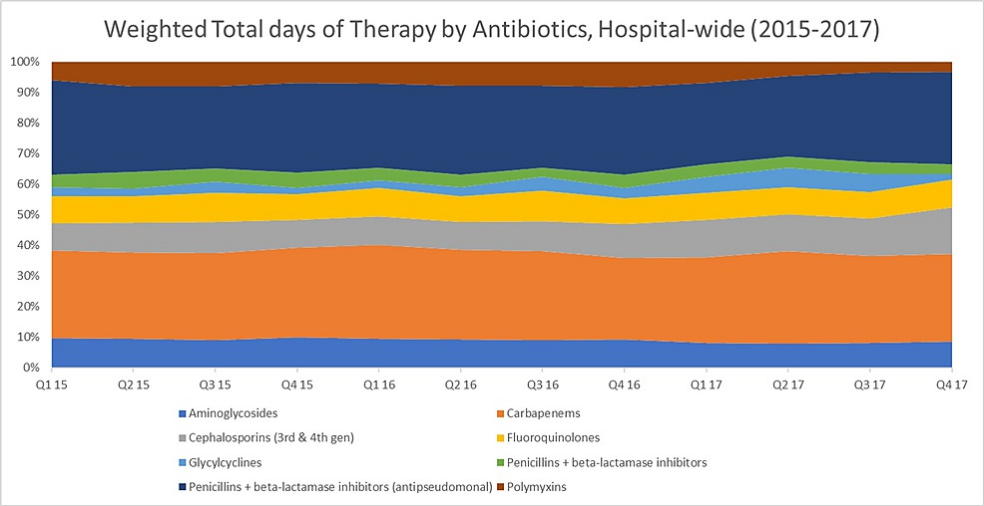
Weighted total days of therapy by antibiotics class, hospital-wide (2015–2017)

**Figure 7:**
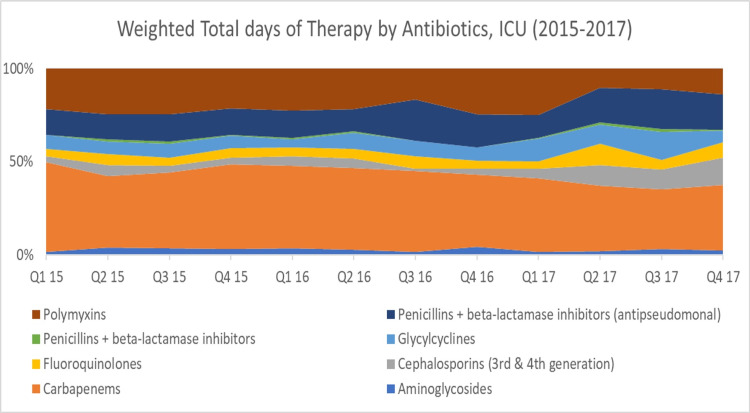
Weighted total days of therapy by antibiotics class, adult ICU (2015–2017)

**Figure 8:**
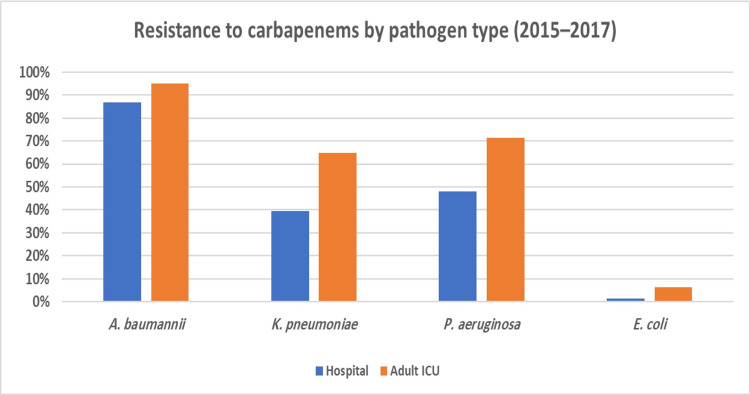
Resistance to carbapenems by pathogen type, hospital-wide and adult ICU (2015–2017)

**Figure 9:**
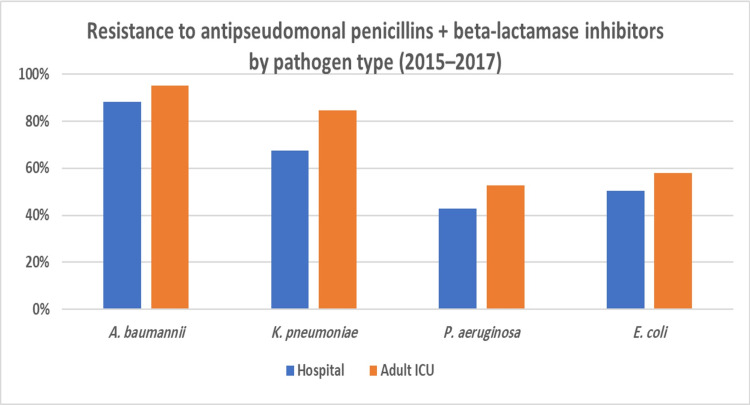
Resistance to antipseudomonal penicillins + beta-lactamase inhibitors by pathogen type, hospital-wide and adult ICU (2015–2017)

Drawing from these results, we explored the rates of antibiotic use and resistance where DRIs were the highest (indicative of low antibiotic effectiveness). Regarding antibiotic consumption, we found that the combined DOT for any antibiotic was much higher in the adult ICU (99.55) as opposed to the entire hospital (47.99). In the adult ICU and across the facility, the top two consumed antibiotic classes were carbapenems and antipseudomonal penicillins + beta-lactamase inhibitors (Figure 6 and Figure 7). However, carbapenems composed a greater proportion of antibiotic prescriptions in the adult ICU (41%) as compared to the facility as a whole (29%). As for resistance to both highly consumed antibiotic classes, *A. baumannii* was the bacteria that had the highest resistance, followed by *K. pneumoniae* and *P. aeruginosa* (Figure 8 and Figure 9). Out of the four bacteria studied, *E. coli* was the least resistant bacteria.

To evaluate the effectiveness of implementing an antibiotic restriction form in the ICU on antibiotic use and resistance, we examined changes in the DRI before and after the intervention was introduced in July 2016 by comparing the mean DRI value in the pre-intervention phase to the mean DRI value in the post-intervention phase. Based on our results (Table 1), the DRI for carbapenems was significantly lower in the post-intervention phase, decreasing from 31.61 to 26.05 (p = 0.031). The DRI for antipseudomonal penicillins + beta-lactamase inhibitors was significantly higher in the post-intervention phase, increasing from 1.24 to 2.37 (p = 0.007). *Acinetobacter baumannii* was affected the most by the reduction of carbapenem consumption in the ICU, with a significant reduction in its resistance to carbapenems in the post-intervention period, as shown in Table 2 (p = 0.021).

**Table 1:** Comparison of the hospital-wide and adult ICU DRI before and after antibiotic restriction

	Hospital-wide			Adult ICU		
	Pre-intervention	Post-intervention		Pre-intervention	Post-intervention	
	Mean (SD)	Mean (SD)	p	Mean (SD)	Mean (SD)	p
Overall DRI	47.11 (1.92)	48.81 (4.12)	0.381	57.46 (5.39)	61.46 (4.06)	0.176
Organism						
Acinetobacter baumannii	80.60 (3.45)	76.59 (3.03)	0.058	72.54 (2.61)	72.04 (5.11)	0.834
Klebsiella pneumoniae	50.81 (5.73)	52.52 (6.74)	0.647	59.70 (8.89)	65.21 (5.12)	0.217
Pseudomonas aeruginosa	41.72 (2.41)	41.80 (5.53)	0.973	51.07 (5.20)	54.37 (7.07)	0.379
Escherichia coli	27.68 (2.30)	30.57 (2.67)	0.072	7.15 (7.93)	19.65 (13.19)	0.075
Antibiotic class						
Aminoglycosides	2.50 (0.33)	2.39 (0.50)	0.656	1.21 (0.34)	1.12 (0.61)	0.764
Carbapenems	11.74 (0.86)	11.64 (1.53)	0.897	31.61 (3.49)	26.05 (4.15)	0.031
Cephalosporin	5.22 (0.22)	6.66 (0.81)	0.202	3.24 (1.04)	5.24 (3.46)	0.204
Fluoroquinolones	4.77 (0.32)	4.77 (0.50)	0.898	3.34 (0.70)	4.62 (1.85)	0.144
Glycylcyclines	1.88 (0.29)	2.57 (0.85)	0.091	4.58 (1.21)	5.30 (1.81)	0.435
Penicillins	3.54 (0.46)	3.84 (0.56)	0.371	0.66 (0.42)	0.47 (0.45)	0.468
Antipseudomonal penicillins	16.94 (0.87)	16.87 (0.96)	0.893	10.40 (1.24)	14.13 (2.37)	0.007
Polymyxins	0.51 (0.22)	1.09 (0.59)	0.050	2.42 (1.02)	4.53 (3.13)	0.147

**Table 2:** Carbapenem resistance comparison before and after antibiotic restriction in the adult ICU

	Hospital-wide			Adult ICU		
	Pre-intervention	Post-intervention		Pre-intervention	Post-intervention	
	Mean (SD)	Mean (SD)	p	Mean (SD)	Mean (SD)	p
Carbapenem resistance (average)	40.34 (2.98)	40.92 (5.95)	0.836	72.66 (6.08)	69.62 (5.23)	0.375
Organism						
Acinetobacter baumannii	88.76 (3.84)	84.92 (4.53)	0.145	97.23 (1.92)	93.01 (3.26)	0.021
Klebsiella pneumoniae	40.22 (9.32)	38.81 (9.08)	0.795	67.38 (12.34)	62.08 (6.87)	0.380
Pseudomonas aeruginosa	48.37 (3.39)	48.01 (7.15)	0.915	71.81 (7.82)	70.69 (11.50)	0.847
Escherichia coli	1.37 (0.66)	1.67 (0.63)	0.435	7.30 (4.45)	5.19 (5.69)	0.491

## Discussion

The rising problem of antibiotic resistance is a serious threat to healthcare institutions and the general public. Despite evidence that inappropriate use and overconsumption of antibiotics results in increased resistance, the majority of antibiotic prescriptions are still used inappropriately. A meta-analysis by Marquet et al. (2015) to explore the incidence of in-hospital inappropriate empiric antibiotic use in patients and the relationship with patient outcomes reported that up to 79% of general antibiotic consumption for the treatment of severe infections in hospitals is inappropriate [11]. In Saudi Arabia, a recent study of antibiotic consumption found that 66% of carbapenems and piperacillin-tazobactam used in a surgical ward were unjustifiably prescribed with or without culture [12].

To the best of our knowledge, this is the first study in Saudi Arabia to calculate the DRI for a healthcare facility. This is also the first study to apply the DRI in a clinical setting. We calculated the DRI for our hospital as a whole and the adult ICU. The DRI in the adult ICU was calculated separately to evaluate the effectiveness of implementing an antibiotic restriction protocol as part of an antibiotic stewardship program (ASP) to control and rationalize antibiotic consumption. The results from the analysis suggested that antibiotic effectiveness was lower in the adult ICU than in the overall hospital throughout the study period indicated by higher DRI in the adult ICU. *Acinetobacter baumannii*, in particular, had the highest DRI among all organisms in both settings. This was due in part to high rates of antibiotic resistance among *A. baumannii*, which in previous studies have also been shown to be the most resistant organisms among the common Gram-negative bacteria with the highest DRI [13].

As far as the DRI by antibiotic class is concerned, in the adult ICU, carbapenems had the lowest antibiotic effectiveness, followed by antipseudomonal penicillins + beta-lactamase inhibitors. For the overall hospital, this finding was reversed. High DRIs and, in effect, low antibiotic effectiveness among carbapenems and antipseudomonal penicillins + beta-lactamase inhibitors upon further analysis appeared to be driven by high rates of consumption of these antibiotic classes.

It seems that carbapenems are the drug of choice for ICU physicians, as many studies have reported the same results. A study conducted by Balkhy et al. (2018) showed carbapenems to be the highest consumed antibiotic class in the adult ICUs of a tertiary care hospital in Saudi Arabia [14]. Likewise, studies in Canada, Australia, and New Zealand in ICUs also demonstrated carbapenems and antipseudomonal penicillins + beta-lactamase inhibitors to be the most consumed antibiotics [15,16].

The results of our study suggested a possible association between the reduction of carbapenem consumption in the adult ICU and decreased carbapenem resistance among *A. baumannii* infections. On the other hand, higher consumption of antipseudomonal penicillins in the adult ICU leads to higher DRI. Antipseudomonal penicillins were not included in the list of restricted antibiotics, so it was easier for the physicians to switch from carbapenems to antipseudomonal penicillins without any restriction, leading to their higher consumption.

Our study also had some limitations. First, we did not have separate data on clinical areas other than the adult ICU to have a clearer comparison of the DRI. The data on the adult ICU was also included in the hospital-wide results. At least having separate data from the pediatric ICU would give us an opportunity to have a fair comparison of the DRI between ICUs. Second, we did not ascertain the appropriateness of antibiotic use to check what percentage of antibiotics was used judiciously. The results of our study are statistically significant in a way that it highlights the importance of having effective antimicrobial stewardship programs in healthcare settings and regular feedback of antibiotic consumption data to the stakeholders to keep the antibiotic prescriptions in check and ensure their sustained effectiveness.

## Conclusions

The results of our study suggested a possible association between the reduction of carbapenem consumption in the adult ICU and decreased carbapenem resistance among *A. baumannii* infections. On the other hand, higher consumption of antipseudomonal penicillins in the adult ICU leads to higher DRI. Antipseudomonal penicillins were not included in the list of restricted antibiotics, so it was easier for the physicians to switch from carbapenems to antipseudomonal penicillins without any restriction, leading to their higher consumption. Further research is required in this field.
